# Dynamic role of the correlation effect revealed in the exceptionally slow autodetachment rates of the vibrational Feshbach resonances in the dipole-bound state†

**DOI:** 10.1039/d1sc05481c

**Published:** 2022-02-04

**Authors:** Do Hyung Kang, Jinwoo Kim, Sang Kyu Kim

**Affiliations:** Department of Chemistry, KAIST Daejeon 34141 Republic of Korea sangkyukim@kaist.ac.kr

## Abstract

Real-time autodetachment dynamics of the loosely bound excess electron from the vibrational Feshbach resonances of the dipole-bound states (DBS) of 4-bromophonoxide (4-BrPhO^−^) and 4-chlorophenoxide (4-ClPhO^−^) anions have been thoroughly investigated. The state-specific autodetachment rate measurements obtained by the picosecond time-resolved pump-probe method on the cryogenically cooled anions exhibit an exceptionally long lifetime (*τ*) of ∼823 ± 156 ps for the 11′^1^ vibrational mode of the 4-BrPhO^−^ DBS. Strong mode-dependency in the wide dynamic range has also been found, giving *τ* ∼ 5.3 ps for the 10′^1^ mode, for instance. Though it is nontrivial to get the state-specific rates for the 4-ClPhO^−^ DBS, the average autodetachment lifetime of the 19′^1^20′^1^/11′^1^ mode has been estimated to be ∼548 ± 108 ps. Observation of these exceptionally slow autodetachment rates of vibrational Feshbach resonances strongly indicates that the correlation effect may play a significant role in the DBS photodetachment dynamics. Fermi's golden rule has been invoked so that the correlation effect is taken into account in the form of the interaction between the charge and the induced dipole where the latter is given by the polarizable counterparts of the electron-rich halogenated compound and the diffuse non-valence electron. This report suggests that one may measure, from the real-time autodetachment dynamics, the extent of the correlation effect contribution to the stabilization and/or dynamics of the excess non-valence electron among many different types of long-range interactions of the DBS.

## Abstract 2

Exceptionally slow autodetachment dynamics of the vibrational Feshbach resonances found in the dipole-bound state of 4-bromophonoxide (4-BrPhO^−^) or 4-chlorophenoxide (4-ClPhO^−^) anions reveals the associated dynamic role of the correlation effect.

## Graphical abstract

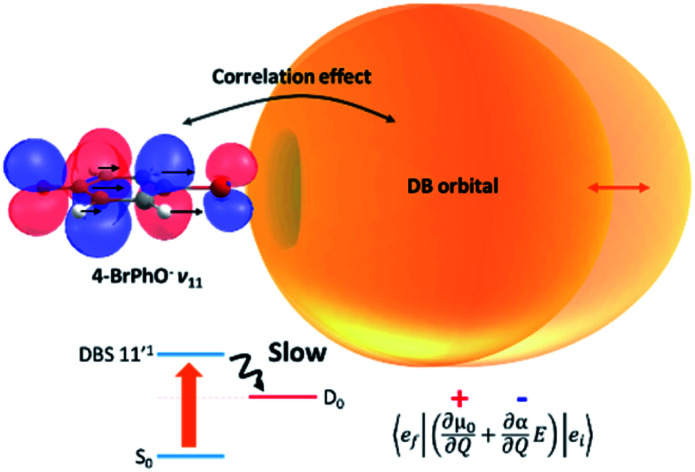

## Introduction

Since firstly conceived by Fermi and Teller,^1^ the dipole-bound state (DBS) of an anion, where the excess electron is loosely bound to the neutral core by the long-range monopole–dipole interaction, has been intensively investigated both experimentally and theoretically in recent decades.^2–9^ The DBS is known to play an important role as the doorway state to stable valence anion formation.^10–14^ Specifically, as the slow electron approaches the neutral molecule or radical, the incoming electron is captured in the form of the Feshbach resonances by the long-range attractive interaction potential, and it is followed by the subsequent coupling and/or relaxation into the more stable anion species.^15,16^ Detailed pictures of the whole processes of the electron-capturing, coupling, and relaxation are thus essential for the thorough understanding of anion chemistry as well as the entry/exit dynamics of the redox reactions. The DBS has been found to be ubiquitous and identified in a number of chemical and biological systems to date. Notably, thanks to the combined techniques of laser spectroscopy and cryogenically cooled ion-trapping,^17^ the understanding of the DBS has recently been enormously advanced in terms of the precise information of energetics, vibrational structures, and state-specific autodetachment (or relaxation) dynamics.^5,18^ Regarding dynamics, however, the state-specific autodetachment rate of the DBS was directly measured only recently for the phenoxide (PhO^−^) anion,^19^ allowing for the stringent comparison of the experiment with the theoretical prediction. Fermi's golden rule has been found to be extremely useful to explain the strongly mode-dependent autodetachment rates although the prediction of the absolute values seems to be still quite challenging.^19^

The lower threshold of the dipole moment for holding the excess electron was firstly proposed to be 1.625 D,^20^ although it has been refined repeatedly after the correction of the Born–Oppenheimer approximation,^21,22^ for instance. More practically, however, it seems to be widely accepted now, as a rule of thumb, that the DBS may exist when the dipole moment of the neutral core exceeds 2.5 D.^23,24^ Although the long-range interaction of the dipole moment of the neutral core with the excess non-valence electron has been considered to be the most critical factor in the electron binding/unbinding dynamics, many theoretical studies have suggested that the (especially dispersive) electron correlation effect should be largely responsible for the excess electron binding to the neutral core in the DBS.^4,25–27^ The significant contribution of the electron correlation effect to the binding energy of the DBS has been theoretically demonstrated by quantum-mechanical calculations using the 2nd-order Møller–Plesset perturbation (MP2), the coupled cluster singles and doubles (CCSD) theory, or the quantum Monte Carlo method.^28–31^ In the same context, it is notable that the critical value of the dipole-moment for the existence of the π-type DBS is still in dispute.^32–34^ Although the extent of the correlation effect is highly anticipated to be strongly dependent on individual chemical systems, the importance of the correlation effect in the DBS seems to be well received in the scientific community. Apparently, however, it is nontrivial to experimentally identify the correlation effect in terms of the static and/or dynamic role in the DBS. Even though the electron binding energy of the DBS is often expected to be proportional to the dipole-moment magnitude of the neutral core, it does not necessarily mean that the electron binding of the DBS is governed by the dipole moment only, as many different factors related to the long-range interaction potential could be cancelled out or added up depending on the chemical details.^35^ For example, the smaller (or lager) binding energy does not necessarily mean the smaller (or larger) contribution of the correlation effect, and *vice versa*.

In this aspect, we have here found that the correlation effect may be reflected in the dynamic property of the DBS rather than in the static binding energy. For example, two different DBS chemical systems of similar binding energies could be quite different in terms of the extent of the correlation-effect. Herein, we argue that the autodetachment dynamics could reveal the nature of the electron binding in terms of the dynamic role of the correlation effect in the electron binding/unbinding dynamics of the DBS. We have investigated the picosecond (ps) time-resolved autodetachment dynamics of the DBS vibrational Feshbach resonances prepared by the one-photon photoexcitation of the cryogenically-cooled 4-bromophenoxide (4-BrPhO^−^) and 4-chlorophenoxide (4-ClPhO^−^) anions. The exceptionally slow autodetachment dynamics observed in some vibrational Feshbach resonances of these anions have been analyzed from the new perspective that the correlation effect may play a significant role in the autodetachment dynamics.

## Methods

Details of the electrospray ionization-photoelectron imaging (ESI-PEI) apparatus have been described elsewhere.^36^ 1 mM concentration of the phenol and 4-XPhOH (X = -Cl, -Br, TCI chemicals Inc,) were dissolved in a 9 : 1 methanol/water solution without further purification. Anions were generated from the home-made ESI source and de-solvated by the dual-stage ion funnel (IF141, Masstech Inc.). Target anions were transferred into the cryogenic (∼8 K) Paul ion trap where they were internally cooled with the buffer gas of the (4 : 1) He/H_2_ mixture. The cryogenically cooled anions were then extracted and accelerated to the velocity map electron imaging (VMI) apparatus. Photoelectrons ejected by the interaction with the picosecond laser pulses were detected by the microchannel plates (MCP) backed by the phosphor screen prior to being recorded by a charge-coupled device (CCD) camera. Picosecond laser pulses were generated from the picosecond Ti:sapphire regenerative amplifier (Legend Elite-P, Coherent) seeded by the femtosecond Ti:sapphire oscillator (Vitara-T-HP, Coherent). One half of the fundamental output of the regenerative amplifier was tuned by an optical parametric amplifier (TOPAS-800, Light Conversion) for the pump laser pulse, while the other half of the fundamental was used as the probe laser pulse. Time-resolved photoelectron images were obtained by scanning the delay times between the pump and probe pulses using a couple of retro-reflectors on the optical delay stage (DDS220, Thorlabs). Photoelectron images were reconstructed by the BASEX^37^ or polar onion peeling (POP) programs.^38^

## Results and discussion

**Fig. 1:**
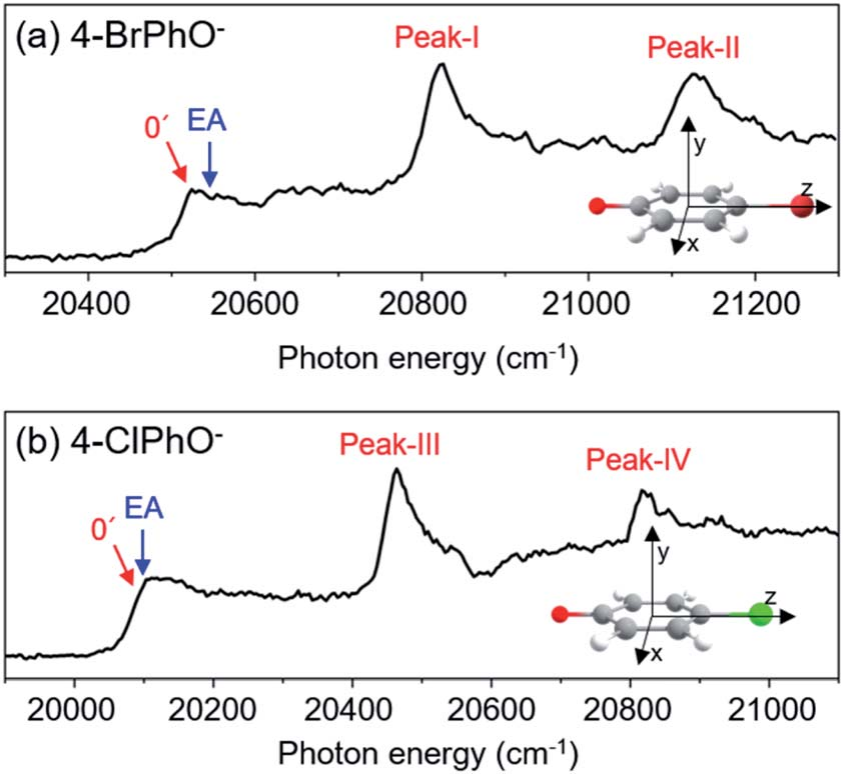
Picosecond photodetachment spectra of (a) 4-BrPhO^−^ and (b) 4-ClPhO^−^. Vibrational states of the DBS are labeled in red while the electron affinity (EA) of each anion is labeled by the blue arrow. Cartesian coordinates of each anion are depicted in the insets.

Photodetachment spectra of the cryogenically-cooled 4-BrPhO^−^ and 4-ClPhO^−^ anions taken by monitoring the total photoelectron signal as a function of the pump laser wavelength are shown in Fig. 1. In both spectra, the stepwise increases of the photoelectron signal represent the electron-affinity (EA) thresholds. The rather sharp peaks are attributed to the vibrational Feshbach resonances of the DBS whereas the diffusive structureless background signal originates from the direct photodetachment of the anion. The overall structures of the photodetachment spectra are more or less identical to those obtained by the nanosecond (ns) laser pulse reported by the Wang group^23^ except for the broad bandwidths of the vibrational bands due to the intrinsic property of the picosecond laser pulse (Δ*E* ∼ 20 cm^−1^, Δ*t* ∼ 1.7 ps). The binding energy of the DBS has been precisely estimated to be 24 or 11 cm^−1^ for 4-BrPhO^−^ or 4-ClPhO^−^, respectively.^23^ The zero-point energy (ZPE) level of the DBS is hardly identified, as the ps laser bandwidth is comparable to the electron binding energy for both anions. Instead, the 11′^1^/20′^1^30′^1^ (peak-I) and 11′^2^/10′^1^ (peak-II) bands could be well identified for 4-BrPhO^−^, whereas the 19′^1^20′^1^/11′^1^ (peak-III) and 11′^1^19′^1^20′^1^/11′^2^ (peak-IV) bands stand out in the DBS of 4-ClPhO^−^, Fig. 1. Compared to the previously reported highly-resolved spectra, the individual DBS vibrational modes could not be spectroscopically resolved in the broad bands in this work.

**Fig. 2:**
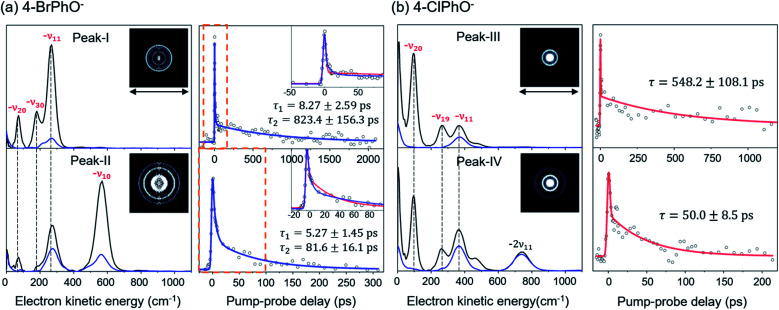
Photoelectron spectra and time-resolved photoelectron transients of the (a) 4-BrPhO^−^ and (b) 4-ClPhO^−^ DBS. (Left panels in (a) and (b)) photoelectron spectra (black) taken at individual vibrational Feshbach resonances are compared with those taken at the adjacent non-resonant pump wavelengths (blue). Raw spectra of the latter are shifted by the amount of the photon energy difference for the purpose of the comparison. Largely-enhanced photoelectron peaks at the resonant excitations are appropriately labeled (red) by the vibrational modes responsible for the autodetachment. Reconstructed photoelectron images at the vibrational resonances are shown in the insets. The pump laser polarization axis is denoted as a black arrow. (Right panels in (a) and (b)) picosecond time-resolved photoelectron transients taken at individual vibrational resonances are fitted by either the single (red) or bi-exponential (blue) decay function. Time constants are extracted from the most reliable fits. For 4-BrPhO^−^, two distinct time constants are obtained from the bi-exponential fits (see the text). Both single and bi-exponential fits are shown in the insets for 4-BrPhO^−^.

Now, by employing the ps pump-probe scheme, we could measure the state-specific autodetachment rates of vibrational Feshbach resonances for both 4-BrPhO^−^ and 4-ClPhO^−^, Fig. 2. The autodetachment rate has been determined by the transient taken by monitoring the low kinetic energy electron as a function of the delay-time between the pump and probe laser pulses. The pump laser wavelength is tuned at the particular DBS vibrational band while the spatially overlapped non-resonant probe laser pulse (791 nm) is given at different delay times. At the zero delay-time, the DBS is most efficiently depopulated to give a spike^19^ with the pump-probe cross-correlation width of ∼2.88 ps. With the increase of the pump-probe delay, the transient signal shows the apparent recovery (which is equivalent to the decay in the transients shown in Fig. 2) due to the autodetachment process, giving the lifetime of the DBS Feshbach resonance from the exponential fit to the experiment.

The peak-I transient of 4-BrPhO^−^ shows the bi-exponential behavior with two distinct lifetimes, Fig. 2. The faster decaying component gives the lifetime (*τ*) of ∼8.3 ± 2.6 ps whereas the lifetime of the slow-decaying component is found to be extremely long, giving *τ* ∼ 823 ± 156 ps. As the fundamental 11′^1^ and 20′^1^30′^1^ combinational modes are expected to be co-excited within the ps pump laser spectral window, two distinct lifetimes are ascribed to the autodetachment of two different vibrational modes. For the appropriate matches between the individual vibrational modes and their associated lifetimes, the time-resolved velocity-map photoelectron images have been taken at the pump wavelength of the peak-I. The photoelectron spectra give the nature of the DBS band as the propensity rule of Δ*v* = −1 is strictly obeyed. Accordingly, in the photoelectron spectrum (Fig. 2) taken from the peak-I, the −*ν*_11_ band represents the autodetachment of the 11′^1^ mode whereas the −*ν*_20_ or −*ν*_30_ photoelectron band is the consequence from the autodetachment of the 20′^1^30′^1^ combination mode *via* the wobbling associated with the *ν*_20_ or *ν*_30_ mode, respectively. The time constants of the 11′^1^ or 20′^1^30′^1^ mode, obtained from the integration over the corresponding distinct energetic windows in the time-resolved photoelectron images (see ESI†) are estimated to be ∼534 ± 87 ps (253–500 cm^−1^ for −*ν*_11_) or ∼61 ± 34 ps (25–130 cm^−1^ for −*ν*_20_), respectively. Although the time constants do not exactly match, this strongly supports that the exceptionally long lifetime of ∼ 823 ps shown in the upper trace of Fig. 2(a) represents the autodetachment from the 11′^1^ mode whereas the faster decaying component with *τ* ∼ 8.3 ps should be ascribed to the autodetachment from the 20′^1^30′^1^ combinational mode. It should be noted that the time constants extracted from the low kinetic energy photoelectron transients taken by the continuous scan of the pump-probe delay time (Fig. 2) are taken to be more reliable here compared to those from the time-resolved photoelectron images as the experimental conditions are hardly kept identical for all images in the latter. Interestingly, whereas the autodetachment lifetime of the 20′^1^30′^1^ mode sounds reasonable in terms of the order of magnitudes,^19^ the lifetime of the 11′^1^ mode seems to be extraordinarily long for the vibrational autodetachment process. It should be noted though that the estimated lifetime of 823 ps of the 11′^1^ mode has a somewhat large uncertainty due to the narrow temporal window (0–2.2 ns) of the present experimental conditions. Nonetheless, it is quite remarkable that the autodetachment rate of the 11′^1^ mode of the 4-BrPhO^−^ DBS is ∼25 times slower compared to that of the 11′^1^ mode of the PhO^−^ of which the lifetime has been measured to be ∼33.5 ps.^19^ Considering that the infrared intensity of the *ν*_11_ mode of the 4-BrPhO is only two times weaker than the *ν*_11_ mode of the PhO (*vide infra*), the retardation of the DBS autodetachment of the former compared to the latter by more than one order of magnitude is quite exceptional. The somewhat similar analysis for the 11′^2^/10′^1^ band (peak-II) of 4-BrPhO^−^ has also been carried out in order to discriminate closely-lying 11′^2^ and 10′^1^ modes (ESI†), giving *τ* ∼ 82 or 5.3 ps for the 11′^2^ or 10′^1^ mode, respectively. The autodetachment rate of the 11′^2^ mode of 4-BrPhO^−^ (*τ* ∼ 82 ps) is also quite slow compared to that of the 11′^2^ mode of PhO^−^ (*τ* ∼ 12 ps),^19^ supporting the experimental finding of the extremely slow autodetachment rate for the 11′^1^ mode of 4-BrPhO^−^.

In order to explain the experiment, we have invoked Fermi's golden rule which has been widely used for the autodetachment rate.^39–42^1
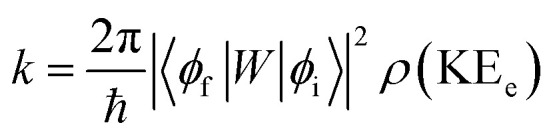
where,2
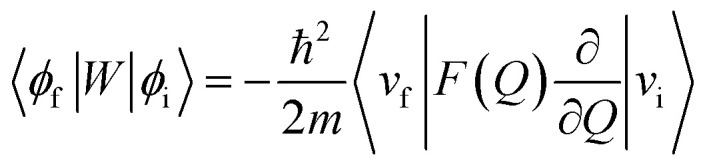
3
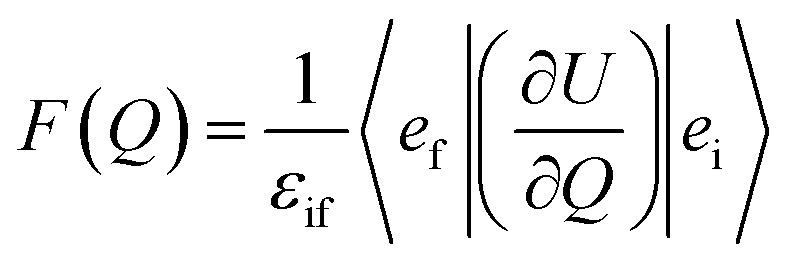


Here, *ϕ*_i_ and *ϕ*_f_ are the initial and final total wavefunctions, respectively, whereas *v*_i_ (*e*_i_) or *v*_f_ (*e*_f_) is the initial or final vibrational (electronic) wavefunction, respectively. *ρ* is the density of states which is the function of the electron kinetic energy (KE_e_). *U* is the charge–dipole interaction potential for the excess electron whereas *Q* is the normal mode coordinate associated with the particular vibrational mode. When the electron binding potential is confined to the interaction between the charge and permanent-dipole moment (*μ*_0_), *F*(*Q*) is proportional to the magnitude of the derivative of *μ*_0_ with respect to *Q*, ∂*μ*_0_/∂*Q*. As the infrared (IR) intensity is proportional to (∂*μ*_0_/∂*Q*)^2^, it is approximately regarded as the quantitative measure of the relative autodetachment rate of the corresponding vibrational mode.^39,43^ Actually, the mode-dependent behavior of the autodetachment rate of the PhO^−^ DBS could be quite successfully explained by the relative IR intensities of the individual vibrational modes as well as the Franck–Condon derivative factor for the overtone band.^19^ In this regard, the more than one order of magnitude increase of the lifetime of the 11′^1^ mode of 4-BrPhO^−^ compared to that of the 11′^1^ PhO^−^ mode cannot be explained by the simple application of the conventional Fermi's golden rule, especially as the IR intensity of the former is only two times weaker than that of the latter (*vide supra*).

It should be emphasized that the autodetachment rate is little influenced by the amount of the electron-binding energy. Rather, the loosely-bound electron is shaken off by the dynamic change of the interaction potential induced by the vibrational wobbling motion.^44^ In this regard, one may invoke the aforementioned electron correlation effect into the autodetachment dynamics for the explanation of the large discrepancy of the experiment from the conventional Fermi's golden rule, especially as the electron-rich halogen atomic moiety is expected to be strongly correlated with the non-valence electron at the positive end of the dipole. Instead of the quantum-mechanical Hamiltonian, we have brought a simple physical model where the interaction potential in Fermi's golden rule is modified to include the interaction between the charge and the (newly-added) induced dipole moment (*μ⃗*_ind_). The effective dipole moment (*μ⃗*_eff_) is then the sum of the permanent and the induced dipole-moments. The induced dipole moment can be expressed by the relation of
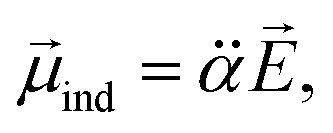
where 
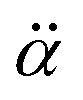
 is the polarizability tensor of the neutral core whereas *E⃑* is the local electric field given by the excess non-valence electron. This may belong to the same context as a recent report by the Wang group that the excess dipole-bound electron may play a significant role as the source of the intramolecular E-field.^45^ The simple physical model proposed here, though the quantum-mechanical correlation effect would be much more sophisticated, may be at least conceptually consistent with the correlation effect, in our opinion, as far as the electron–radical interaction is concerned, as the electrons of the neutral core (although the interaction of the excess electron with all the individual electrons of the neutral core is not taken into account) are allowed to be dynamically correlated with the excess non-valence electron by the mediation of the polarizability and the local electric field. *F*(*Q*) could be then re-written as follows.4
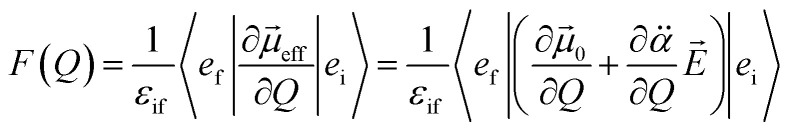


**Fig. 3:**
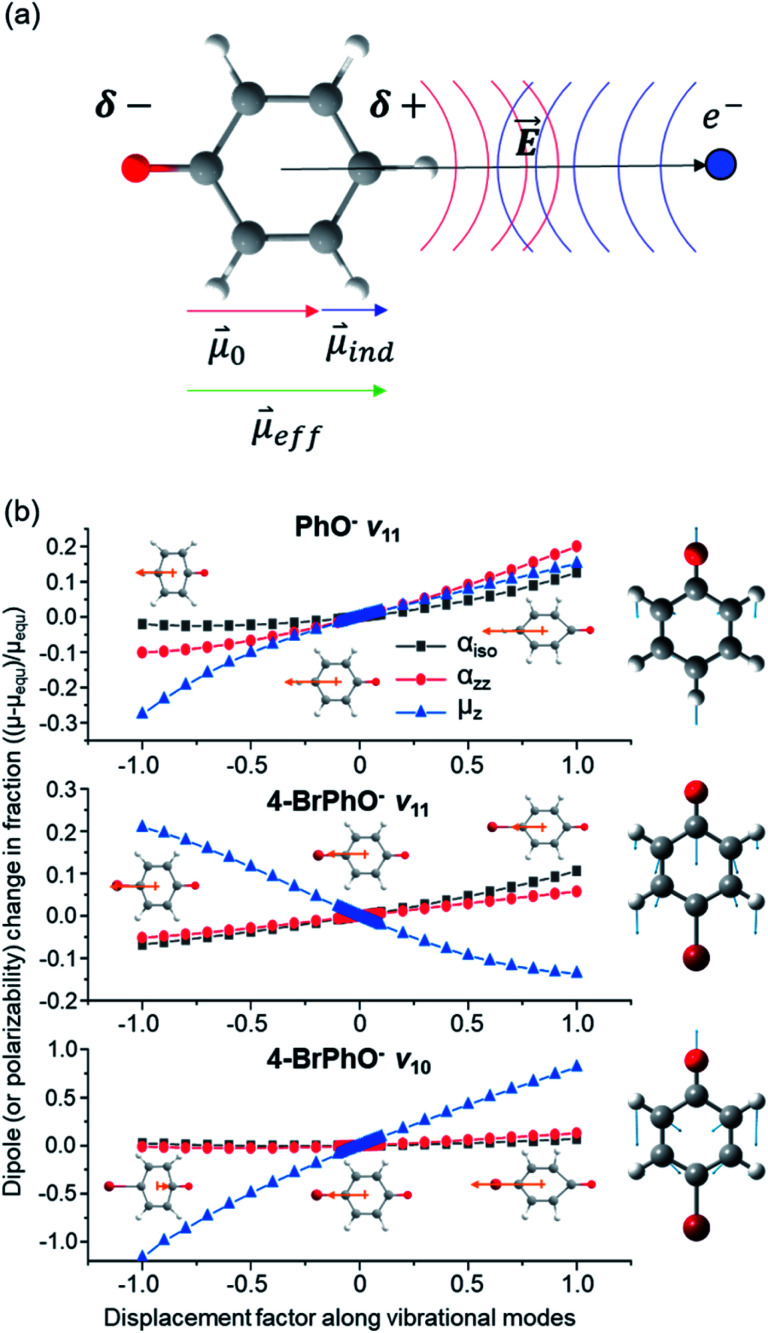
(a) Schematic diagram of the permanent dipole moment (*μ⃗*_0_) and induced dipole moment (*μ⃗*_ind_) in the presence of the electric field (*E⃑*) given by the excess dipole-bound electron. (b) Changes of the dipole moments along the *z*-axis (*μ*_*z*_), those of isotropic polarizabilities (*α*_iso_), or those of polarizabilities along the diagonal *z*-axis (*α*_*zz*_) for the PhO˙ and 4-BrPhO˙ radicals calculated with respect to the *ν*_11_ and *ν*_10_ vibrational modes. Normal mode displacement vectors are denoted in the right panel. Dipole moments (orange arrow) at both ends of the normal mode displacement and equilibrium geometry are denoted. Note that the direction of the dipole moment is denoted according to that of the actual electric dipole moment vector.

Here, *E⃑* is assumed to be independent of *Q*. At the equilibrium position, as *E⃑* heads from the neutral core to the dipole-bound electron, the neutral core is polarized so that the oxygen moiety is negatively charged whereas the opposite-positioned bromine moiety should be positively charged according to 
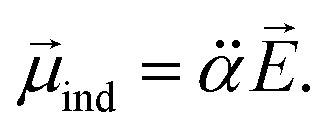
 The resultant induced-dipole, therefore, points the same direction as the permanent dipole (Fig. 3(a)). And yet, the autodetachment process is not determined by the static property of the dipole. Rather, it is governed by the dynamic interplay between the instant changes of the permanent- and induced-dipoles with respect to the particular vibrational normal mode (*Q*). Accordingly, the directions of ∂*μ*_0_/∂*Q* and 
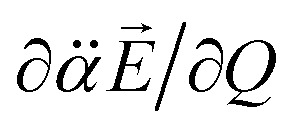
 vectors (the positive or negative slope with respect to *Q*) determine whether or not the correlation effect expedites or impedes the autodetachment process. Namely, if both ∂*μ*_0_/∂*Q* and 
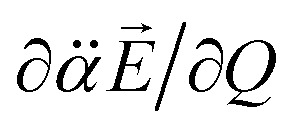
 have the positive (or negative) slopes with respect to *Q*, then the autodetachment rate would increase, indicating that the correlation effect facilitates the autodetachment process. And yet, if the slope of ∂*μ*_0_/∂*Q* is positive (or negative) while the slope of 
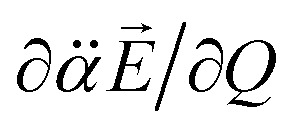
 is negative (or positive), then the magnitude of the vector sum diminishes to give the decrease of the autodetachment rate. In this case, the autodetachment should be retarded due to the correlation effect. It should be noted that, when the dipole-bound electron is regarded as a point-charge lying on the molecule-fixed *z*-axis (Fig. 1 and 3), all the in-plane vibrational modes of PhO^−^ or 4-BrPhO^−^ end up with the instant changes of dipole moments along the *z*-axis.

In order to verify the physical model, we have calculated the ∂*μ*_0_/∂*Q* and ∂*α*/∂*Q* terms for the *ν*_11_ modes of 4-BrPhO and PhO, Fig. 3(b). For ∂*μ*_0_/∂*Q*, *μ*_0_ is identical to the *z*-component of the permanent dipole moment (*μ*_*z*_) (*vide supra*). Regarding ∂*α*/∂*Q*, the derivative of the isotropic polarizability (*α*_iso_) or that of the polarizability along the molecular *z*-axis (*α*_*zz*_) has been separately calculated with respect to the *ν*_11_ mode coordinate, and thus we denote *α* as either *α*_iso_ or *α*_*zz*_. It should be emphasized again that the *ν*_11_ modes of two different neutral cores of 4-BrPhO and PhO have their own normal-mode characteristics in terms of the detailed nuclear displacements. Remarkably, whereas the signs of ∂*μ*_0_/∂*Q* and ∂*α*/∂*Q* are the same for the *ν*_11_ mode of PhO, it has been found that the slope of ∂*μ*_0_/∂*Q* has the opposite sign from that of ∂*α*/∂*Q* for the *ν*_11_ mode of 4-BrPhO. Apparently, the latter is the consequence from the reduction of the permanent dipole moment of 4-BrPhO with the positive displacement of the *ν*_11_ mode whereas the polarizability along the *z*-axis instantly increases by the same displacement (Fig. 3). Substitution of the electronegative Br atom on the *para* position should be responsible for the opposite behavior of the dipole-moment change with *ν*_11_, compared to that of PhO. Therefore, it is most likely that the correlation effect embodied in the charge-induced dipole interaction should impede the autodetachment of the 11′^1^ mode of 4-BrPhO^−^ whereas it expedites that of the 11′^1^ mode of PhO^−^. Though the quantitative comparison is nontrivial, it gives the rational explanation why the autodetachment rate could be exceptionally slow for the 11′^1^ mode of 4-BrPhO^−^. Notably, the magnitude of ∂*α*/∂*Q* could be larger for 4-BrPhO^−^ compared to that of PhO^−^ because of the lager polarizability of the former than the latter although the more sophisticated calculation is highly desirable (ESI†).

The fast autodetachment rate (*τ* ∼ 8.3 ps) observed for the 20′^1^30′^1^ mode of 4-BrPhO^−^ is probably due to the cooperation effect of the combination mode in the wobbling motion as demonstrated previously for PhO^−^ (ESI†).^19^ Regarding the 11′^2^ overtone mode of 4-BrPhO^−^, its lifetime of 82 ps is much longer than the lifetime of 12 ps measured for the 11′^2^ mode of PhO^−^. According to the derivative Franck–Condon factor in eqn (2), the autodetachment rate of the overtone mode is anticipated to be ∼4 times faster than that of the fundamental mode.^19^ In that sense, if the lifetime of the 11′^1^ mode of 4-BrPhO^−^ is taken to be 823 ps, the lifetime of ∼200 ps is expected for the 11′^2^ mode. In the same context, if the lifetime of 82 ps is taken for the 11′^2^ mode, the autodetachment lifetime of the 11′^1^ mode is expected to be ∼330 ps, which is already quite long for the vibrational autodetachment lifetime. Therefore, although the lifetime measurement of 823 ps is subject to the further refinement, it seems to be quite certain that the autodetachment rate of the 11′^1^ mode of 4-BrPhO^−^ is exceptionally slow. The fast autodetachment rate of the 10′^1^ mode of 4-BrPhO^−^ with *τ* ∼ 5.3 ps is mainly attributed to the much stronger IR intensity of the *ν*_10_ mode which is ∼30 times larger than that of *ν*_11_, although Fermi's golden rule could not give the quantitative explanation of the experiment (ESI†). It is interesting to note that the magnitude of ∂*α*/∂*Q* is much smaller than that of ∂*μ*_0_/∂*Q* for the 10′^1^ mode of 4-BrPhO^−^, Fig. 3, suggesting that the dynamics of the corresponding mode is little influenced by the correlation effect.

Similar analysis has also been carried out for 4-ClPhO^−^. For the peak-III in Fig. 1, the 19′^1^20′^1^/11′^1^ DBS band undergoes the autodetachment process *via* −*ν*_19_ (or −*ν*_20_) from the 19′^1^20′^1^ combination mode whereas the autodetachment from the 11′^1^ mode is responsible for the −*ν*_11_ peak. The relative ratio of the former to the latter in the peak-III is estimated to be 0.86 : 0.14. The peak-IV of 4-ClPhO^−^ (Fig. 1) is assigned to the 11′^1^19′^1^20′^1^/11′^2^ band. In the photoelectron spectrum, the photoelectron peak populated by the autodetachment *via* the *ν*_20_ or *ν*_19_ mode is found to be quite small. Similar to the case of peak-III, the relative contribution of the 11′^1^19′^1^20′^1^ and 11′^2^ mode to the peak-IV is estimated to be 0.86 : 0.14. Unfortunately, however, it turns out to be nontrivial to extract two different lifetimes from the transient of peak-III or peak-IV by the bi-exponential fit, mainly due to the relatively poor S/N ratio, Fig. 2. Instead, the single exponential fit to the experiment give the averaged autodetachment lifetime of ∼548 ± 108 or 50.0 ± 8.5 ps for the peak-III or peak-IV, respectively. Overall, the autodetachment rate of the 4-ClPhO^−^ DBS is also estimated to be quite slow compared to that of PhO^−^, indicating that the correlation effect on the electron-binding dynamics could also be quite significant in 4-ClPhO^−^. The overall autodetachment rate of the 4-BrPhO^−^ DBS seems to be slower than that of the 4-ClPhO^−^ 11′^1^, though it should be noted that the autodetachment dynamics is strongly mode-dependent. This could be partially due to the larger polarizability of the former (159.3 Bohr^3^) compared to the latter (138.1 Bohr^3^), though it is subject to the further investigation. It should also be emphasized that the autodetachment dynamics is expected to be strongly dependent on the individual chemical systems, and thus the theoretical analyses for individual chemical systems should be carried out case by case.

**Fig. 4:**
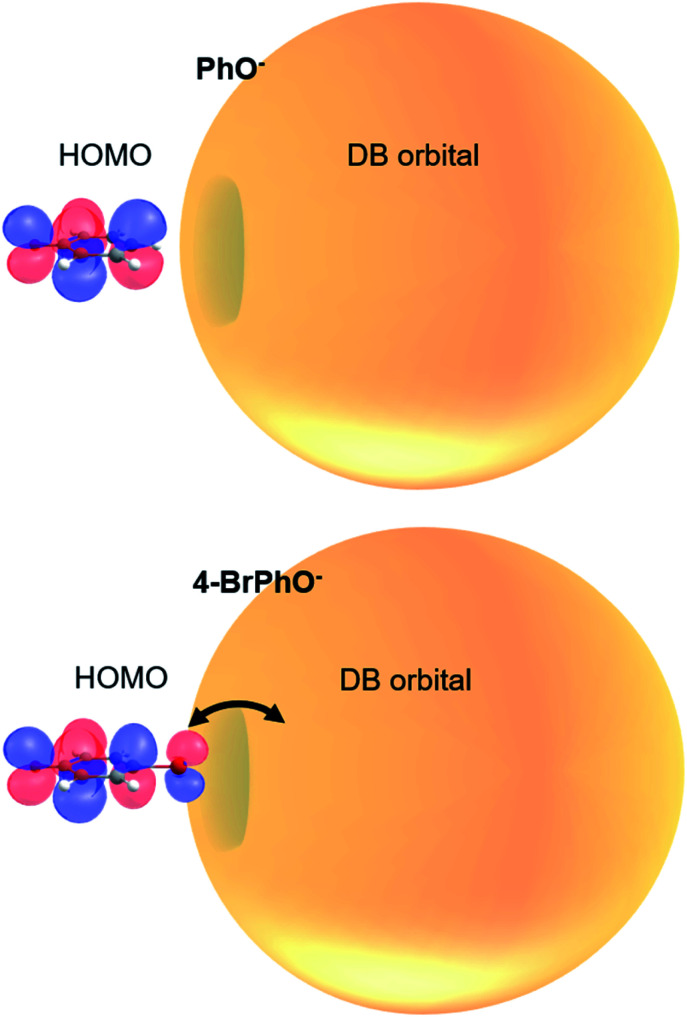
Schematic diagram of the correlation effect between the half-filling HOMO and DB orbital in the DBS electron configuration. Orbital lobes on the Br atom in the *para*-position may enhance the correlation effect between the HOMO and DB orbital, giving the slow autodetachment rate.

It is interesting to note that the highest occupied molecular orbital (HOMO) of the 4-BrPhO^−^ or 4-ClPhO^−^ is delocalized over the entire body including the p-type lobe of the halogen atomic moiety at the positive end of the dipole (Fig. 4). The excess non-valence electron is diffuse and polarizable whereas it could exert the local electric field to influence the electrons in the neutral core.^45^ In this aspect, the relative geometrical orbital arrangements of the neutral-core with respect to the excess electron of which its own arrangement is given by the molecular geometry could be quite critical in the role of the correlation effect in static and dynamic properties of the DBS, which is subject to further investigation in the near future.

## Conclusion

In summary, the conventional Fermi's golden rule based on the simple monopole–dipole interaction potential may not be universal in explaining the autodetachment dynamics of the dipole bound state. Exceptionally slow autodetachment dynamics observed in the DBS of 4-BrPhO^−^ and 4-ClPhO^−^ have been analyzed by adopting the physical model of the induced dipole moment which is conceptually associated with the electron correlation effect as far as the interaction of the excess electron and electrons of the neutral core is concerned. The induced-dipole is dynamic in nature as it is supposed to be the consequence from the local field given by the interaction between polarizable excess non-valence electrons and valence electrons of the neutral-core. A simple physical model, adapted from Fermi's golden rule, turns out to be quite useful in the qualitative explanation of the experiment. The exceptionally slow autodetachment rate of the 11′^1^ mode of 4-BrPhO^−^ DBS may originate from the fact that the derivative of the permanent dipole has the directional property of the opposite sign from that of the polarizability with respect to the corresponding normal mode displacement. In this case, the correlation effect embodied in the physical model impedes the autodetachment process. On the other hand, when the derivative of the permanent dipole and that of the polarizability are the same in their directional properties, the autodetachment is expected to be expedited as demonstrated in the case of the 11′^1^ mode of PhO^−^. This should belong to the case where the autodetachment is facilitated by the correlation effect. The autodetachment process is strongly mode-dependent and also it is highly dependent on the individual chemical system. In this circumstance, the real-time investigation of the autodetachment dynamics, as manifested in this report, could unravel different aspects of the underlying physical principles behind the nature of the non-valence bound states, challenging the sophisticated theoretical analysis in the near future.

## Data availability

The datasets generated and/or analyzed during the current study are available from the corresponding author on request.

## Author contributions

D. H. K. and J. K. conducted whole experiments. D. H. K. performed computations and wrote the paper. S. K. K. conceived the core idea, supervised the whole projects, and edited the manuscript.

## Conflicts of interest

There are no conflicts to declare.

## Supplementary Material

SC-013-D1SC05481C-s001
