# Supplementary material for: Dynamic role of the correlation effect revealed in the exceptionally slow autodetachment rates of the vibrational Feshbach resonances in the dipole-bound state
Source: Chem Sci. 2022 Feb 4;13(9):2714–20. doi: 10.1039/d1sc05481c (PMC8890126; doi:10.1039/d1sc05481c)
Supplement: SC-013-D1SC05481C-s001 [file SC-013-D1SC05481C-s001.pdf]

Electronic Supplementary Material (ESI) for Chemical Science.
This journal is © The Royal Society of Chemistry 2022

Supplementary Information for

Dynamic Role of the Correlation Effect Revealed in the Exceptionally Slow Autodetachment Rates of the Vibrational Feshbach Resonances in Dipole-Bound State

Do Hyung Kang, Jinwoo Kim and Sang Kyu Kim*

Department of Chemistry, KAIST, Daejeon 34141, Republic of Korea

*Corresponding Author: sangkyukim@kaist.ac.kr

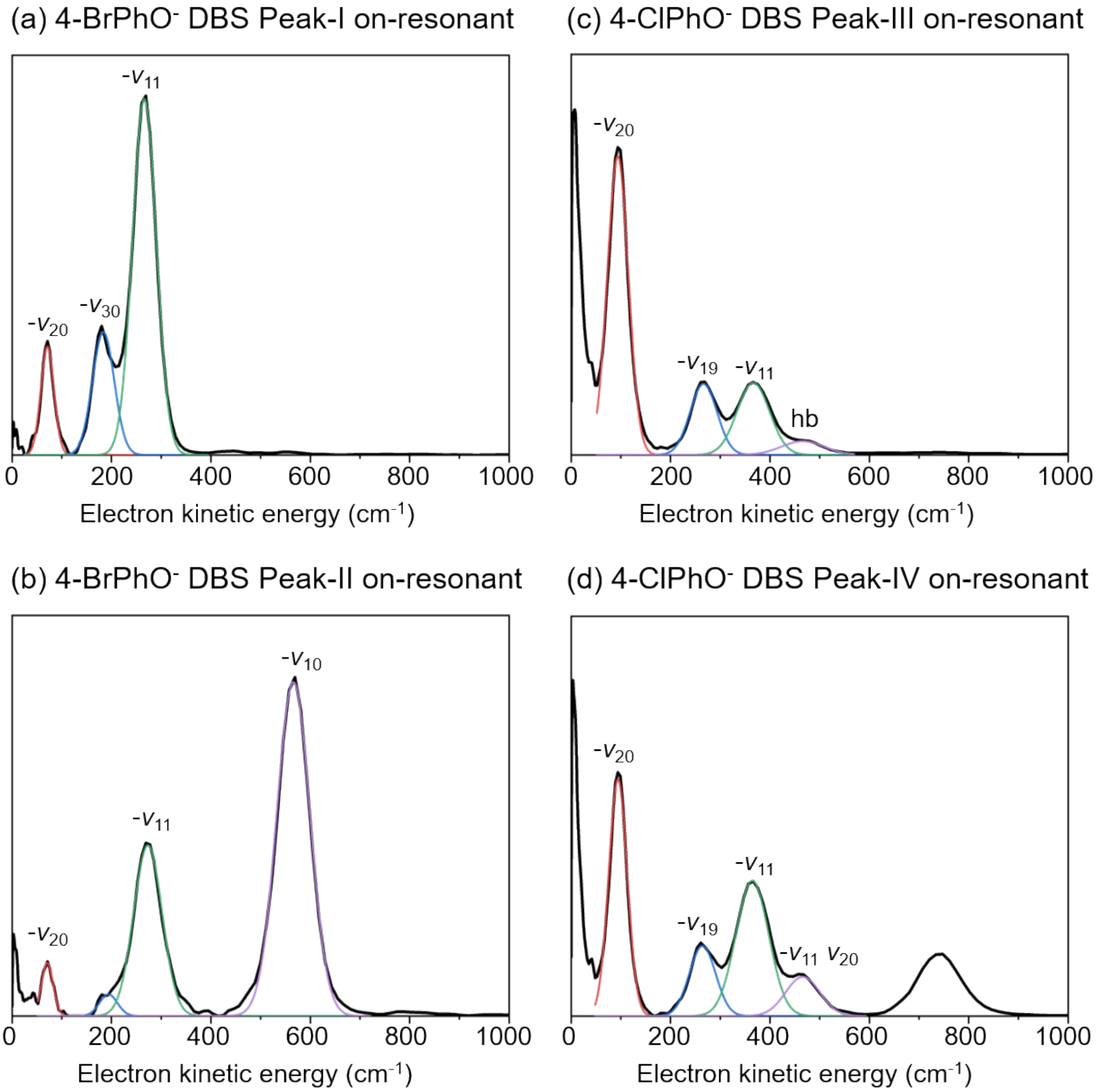

Figure S1. Gaussian deconvolution of the resonant-photoelectron spectra obtained from (a) Peak-I ($11'^{1}/20'^{1}30'^{1}$), (b) Peak-II ($11'^{2}$ (+$10'^{1}$)) of 4-BrPhO$^-$ DBS and (c) Peak-III($19'^{1}20'^{1}/11'^{1}$), (d) Peak-IV ($11'^{1}19'^{1}20'^{1}/11'^{2}$) of 4-ClPhO$^-$ DBS. Detachment from the hot band (hb) is detnoted.

1. Deconvolution of the Resonant/Non-Resonant Photoelectron Imaging

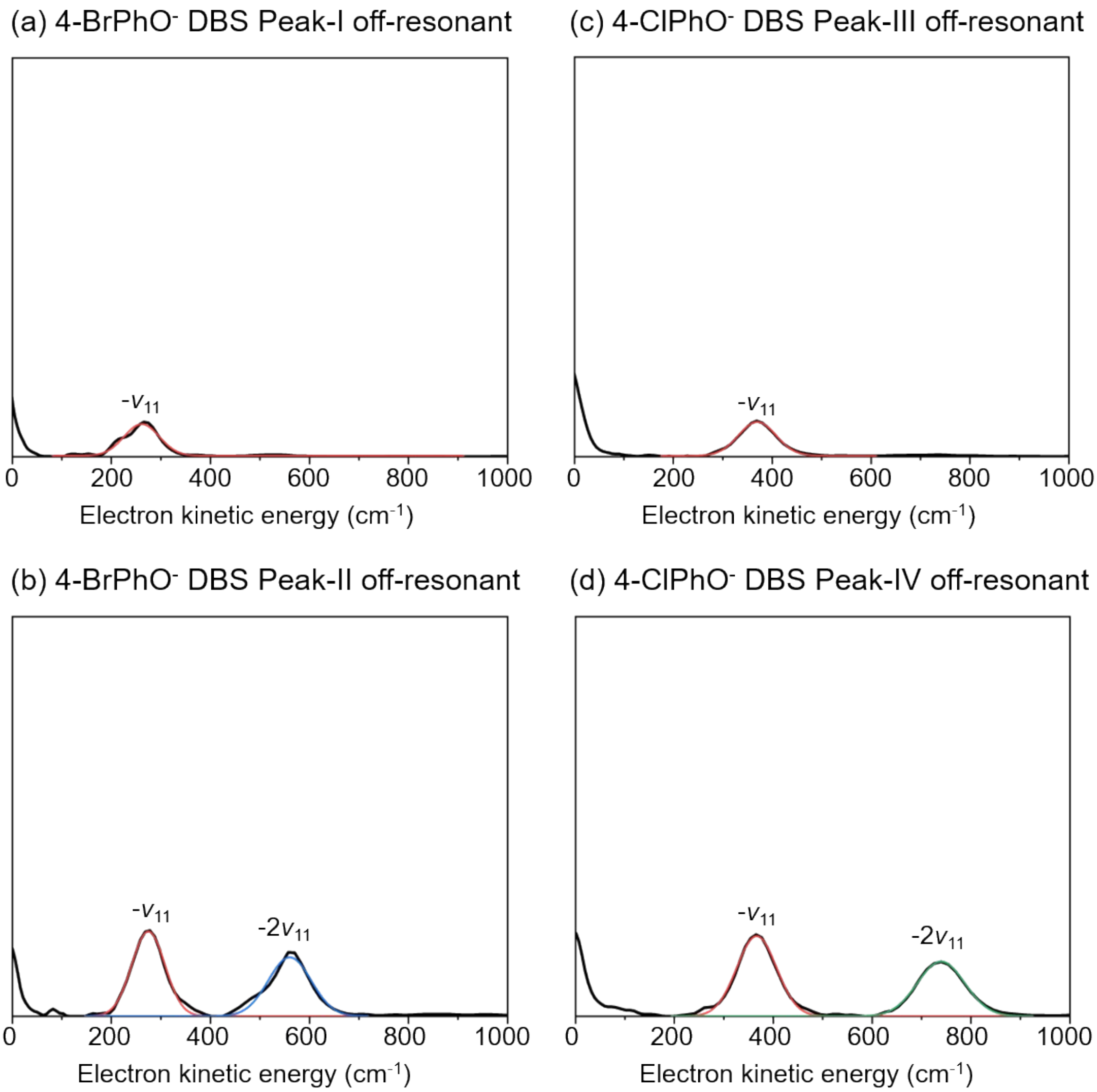

Figure S2. Gaussian deconvolution of the non (off)-resonant photoelectron imaging obtained near the (a) Peak-I ($11'^{1}/20'^{1}30'^{1}$), (b) Peak-II ($11'^{2}$ ($+10'^{1}$)) of 4-BrPhO^{-} DBS and (c) Peak-III($19'^{1}20'^{1}/11'^{1}$), (d) Peak-IV ($11'^{1}19'^{1}20'^{1}/11'^{2}$) of 4-ClPhO^{-} DBS. Each spectra were red-shifted by photon energy discrepancy between resonant and non-resonant photoelectron imaging in order to line up the vibrational peaks.

2. DFT calculation of PhO, 4-BrPhO, and 4-ClPhO

<table>
<tr><th></th><th>PhO
(B3LYP/6-311++g(d,p))</th><th>4-BrPhO
(B3LYP/6-311++g(3df,3pd))</th><th>4-ClPhO
(B3LYP/6-311++g(d,p)</th></tr>
<tr><td>Dipole moment (μ)</td><td>4.0588 Debye</td><td>2.9174 Debye</td><td>2.8135 Debye</td></tr>
<tr><td>Polarizability (α)</td><td>Isotropic (α_{iso}):
72.38 Bohr^3
z-axis (α_{zz}):
99.493 Bohr^3</td><td>Isotropic (α_{iso}):
102.52 Bohr^3
z-axis (α_{zz}):
159.316 Bohr^3</td><td>Isotropic (α_{iso}):
88.60 Bohr^3
z-axis (α_{zz}):
138.111 Bohr^3</td></tr>
<tr><td rowspan="4">Active vibrations</td><td>
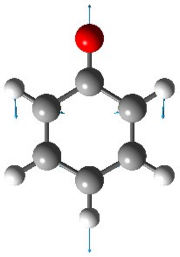
ν_{11} (A_1) 531 cm^{-1}
IR intensity: 2.4575</td><td>
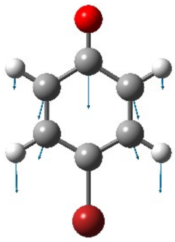
ν_{11} (A_1) 293 cm^{-1}
IR intensity: 1.1036</td><td>
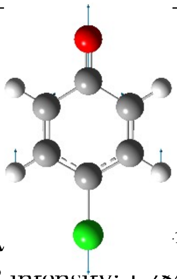
ν [illegible] cm^{-1}
IR intensity: 1.2848</td></tr>
<tr><td></td><td>
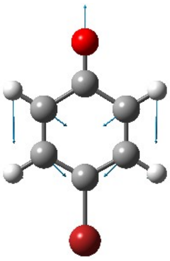
ν_{10} (A_1) 613 cm^{-1}
IR intensity: 36.8049</td><td>
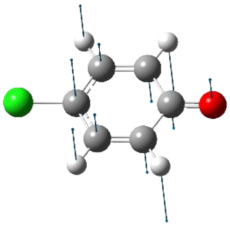
ν_{19} (B_1) 274 cm^{-1}
IR intensity: 3.1519</td></tr>
<tr><td></td><td>
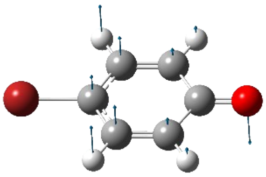
ν_{20} (B_1) 96 cm^{-1}
IR intensity: 4.6074</td><td>
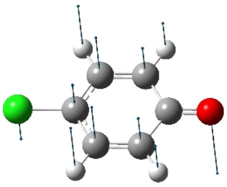
ν_{20} (B_1) 108 cm^{-1}
IR intensity: 5.3676</td></tr>
<tr><td></td><td>
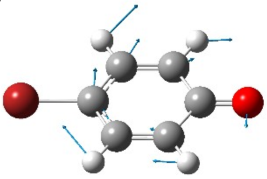
ν_{30} (B_2) 228 cm^{-1}
IR intensity: 2.5924</td><td></td></tr>
</table>

3. Calculation of the Autodetachment from Fermi's Golden Rule

Table S1. Calculated autodetachment rate on each vibrational mode of 4-BrPhO$^-$ by Fermi's golden rule.

Experimentally measured				Calculated			
Transition	eKE (cm^{-1})	ϵ_{if} (cm^{-1})	k^{-1} (ps)	IR intensity	$\partial\mu/\partial Q$ (arb. unit) [a b]	Coupling const [a]	k^{-1} (ps)
$11'^{1}\rightarrow 0_0^0$	271	295	823.4	1.10	1	1	823.4
$11'^{2}\rightarrow 11_0^1$	269	290	81.6	-	-	4.14	200.8
$20'^{1}30'^{1}\rightarrow 30_0^1$	71	95	8.27	4.61	2.05	40.91	34.05
$20'^{1}30'^{1}\rightarrow 20_0^1$	204	228		2.59	1.53	3.94	
$10'^{1}\rightarrow 0_0^0$	584	608	5.27	36.80	5.78	7.86	71.35

[a] Relative constants normalized with respect to the value for the $11'^{1}\rightarrow 0_0^0$ transition.

[b] Derived from the 4-BrPhO radical IR intensity calculation using B3LYP/6-311++g(3df,3pd) method.

4. Dipole moment and polarizability change upon each vibrational mode.

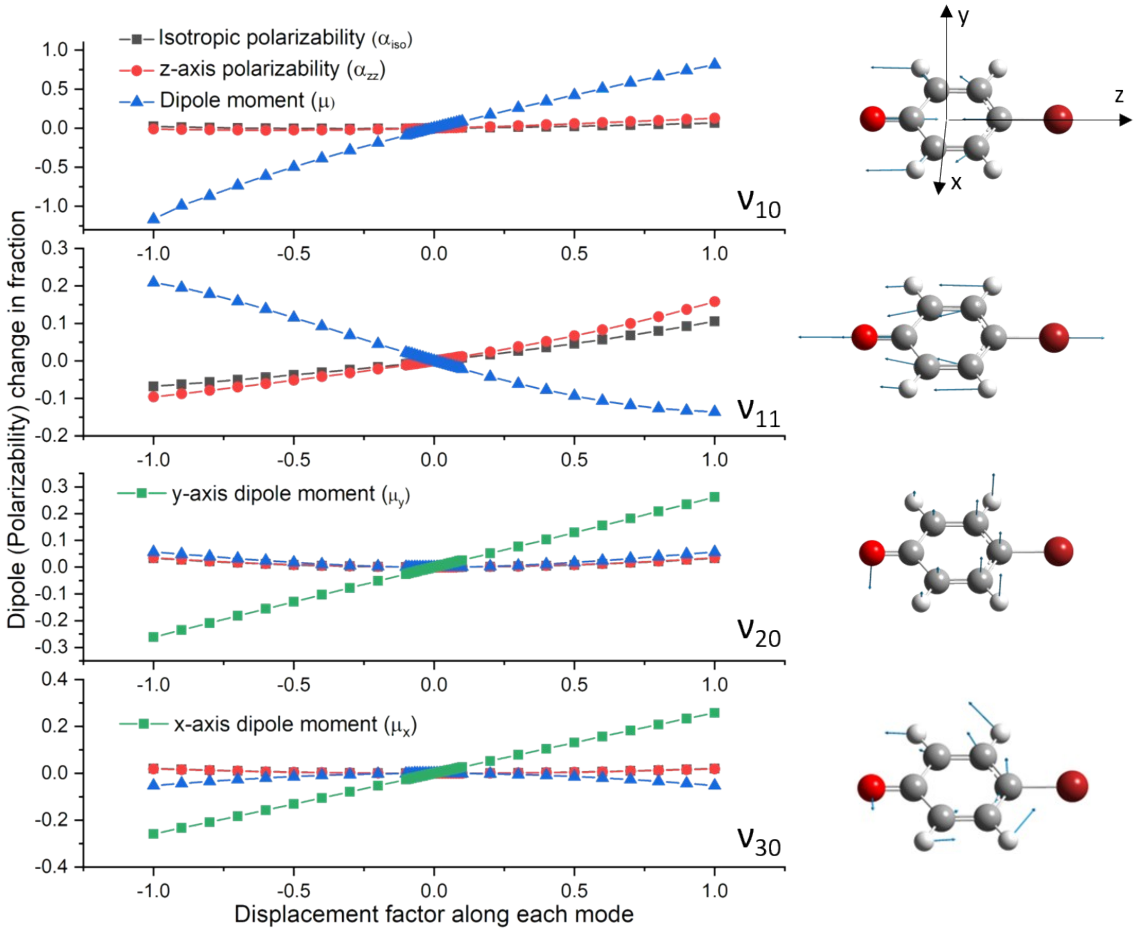

Figure S3. Isotropic polarizabiliy (α_{iso}), z-axis polarizability (α_{zz}), and dipole moment (μ) change in fraction of the 4-BrPhO radical upon each vibrational mode. Displacement vector of each mode is shown in the right panel.

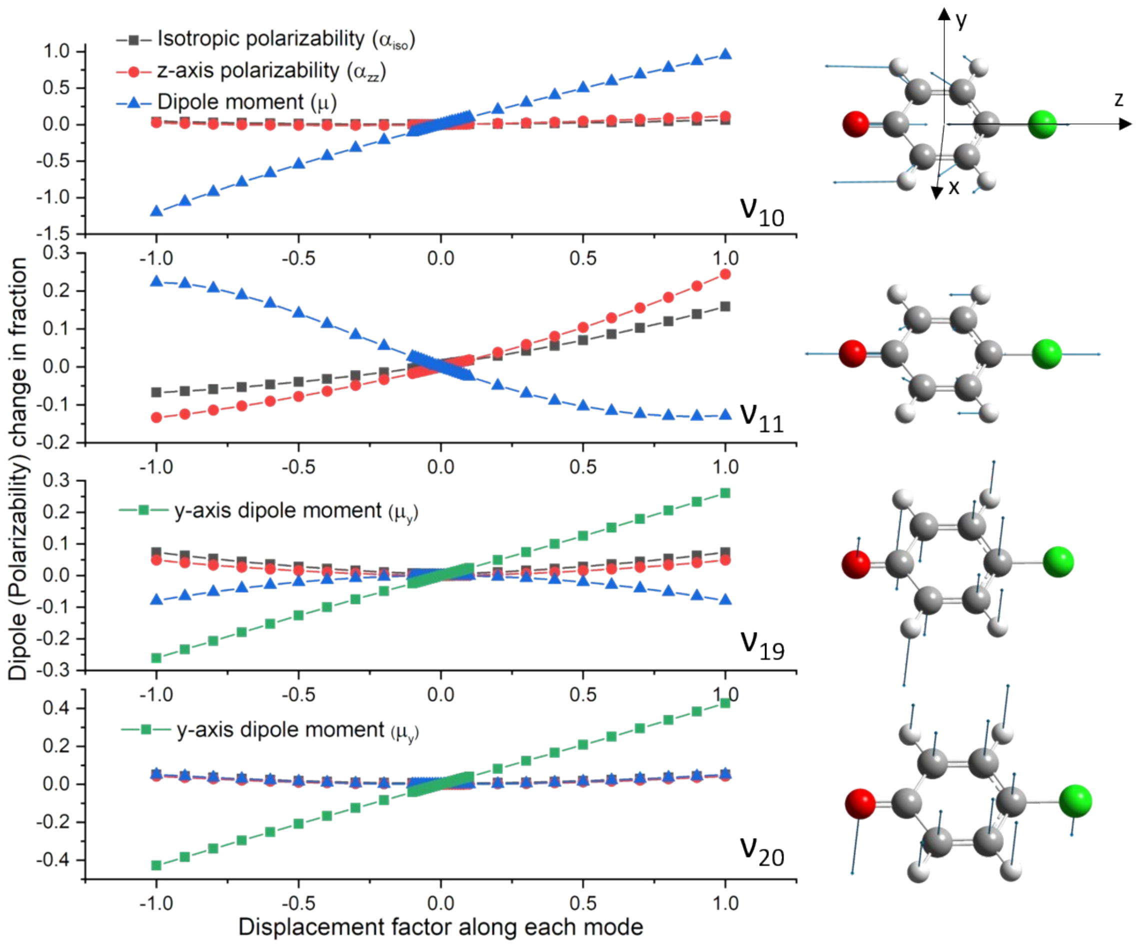

Figure S4. Isotropic polarizability (α_{iso}), z-axis polarizability (α_{zz}), and dipole moment (μ) change of 4-ClPhO radical upon each vibrational mode. Displacement vector of each mode is shown in the right panel.

5. Time-resolved photoelectron imaging (TR-PEI) of 4-BrPhO$^-$ Peak-I

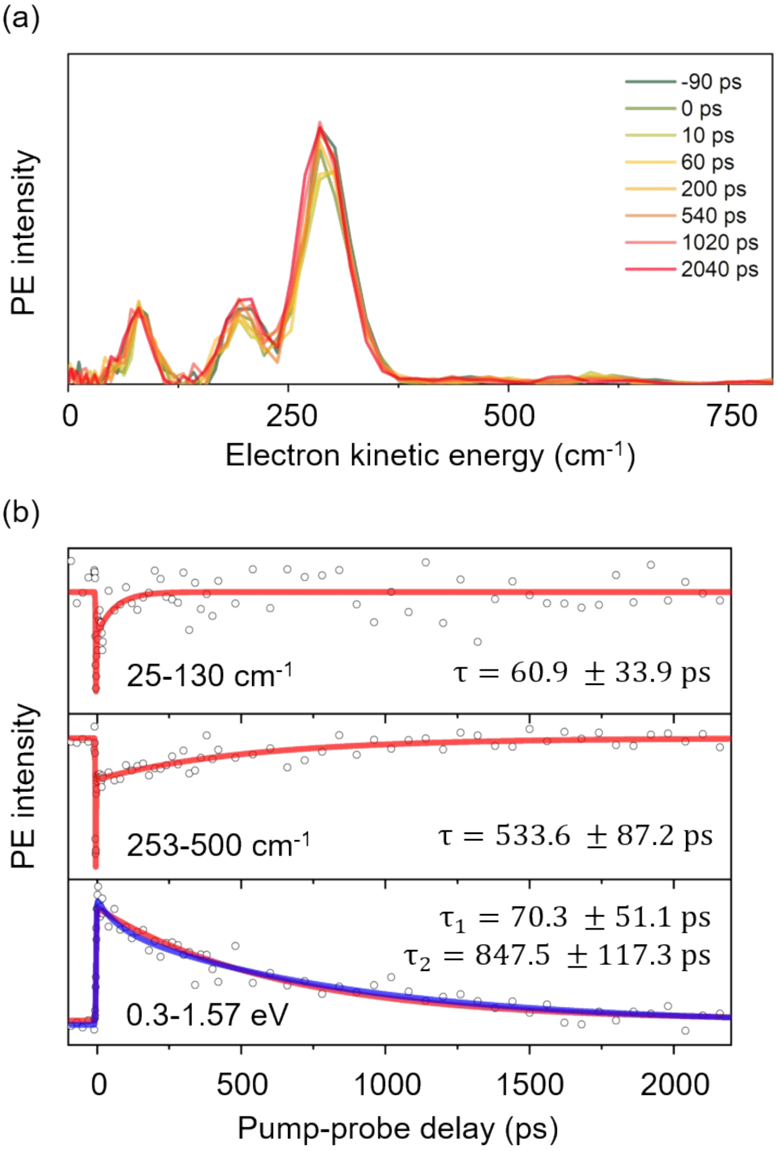

Figure S5. Time-resolved photoelectron imaging (TR-PEI) of 4-BrPhO$^-$ Peak-I ($11'^1/20'^1 30'^1$). (a) Photoelectron spectra of the low-kinetic energy electron (autodetachment) in various pump-probe delay time. (b) Photoelectron transients of the low kinetic energy regions (25-130 cm^{-1} for -ν_{20} and 253-500 cm-1 for -ν_{11}, respectively) and high kinetic energy region (0.3-1.57 eV). Low kinetic energy transients are originated from the autodetachment whereas the high kinetic energy transient is corresponding to the probing of the all the population in DBS by projecting into the high kinetic energy region3

6. Assignment of the 4-BrPhO$^-$ Peak-II

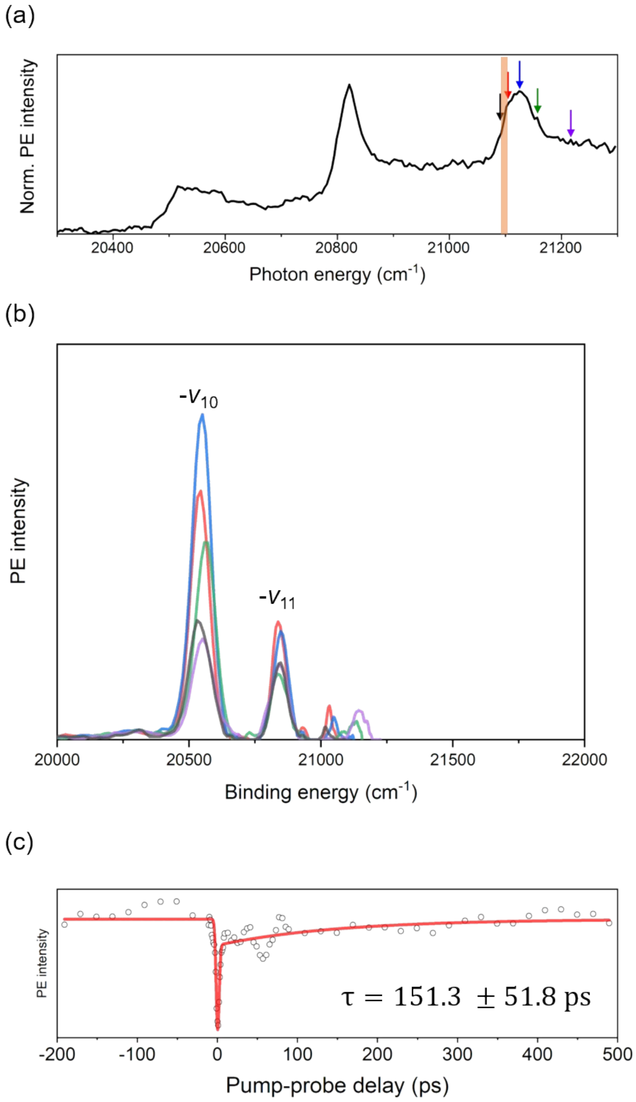

Figure S6. (a) Photodetachment spectrum and (b) photoelectron spectra on the sidebands of the Peak-II. The colors in photoelectron spectra are corresponding to the pump wavelengths on the photodetachment spectrum. Compared to the non-resonant spectrum (purple) and resonant spectrum (blue), spectra obtained in red-side (black and red) showed photoelectron peak enhancement on -ν_{11} than that of the -ν_{10}, while the spectrum from the blue-side band (green) showed enhancement on the -ν_{10} peak. (c) Photoelectron transient obtained at the red-side of the Peak-II (orange shaded area). Slow autodetachment signal was obtained, which possibly originate from the -ν_{11}, corresponding to the autodetachment from the $11'^2$ peak.
